# AIE+ESIPT Active Hydroxybenzothiazole for Intracellular Detection of Cu^2+^: Anticancer and Anticounterfeiting Applications

**DOI:** 10.3390/molecules27227678

**Published:** 2022-11-08

**Authors:** Rajdeep Kaur, Rasdeep Kour, Satwinder Singh Marok, Satwinderjeet Kaur, Prabhpreet Singh

**Affiliations:** 1 Department of Chemistry, UGC Centre for Advanced Studies-II, Guru Nanak Dev University, Amritsar 143001, India; 2 Department of Botanical and Environmental Sciences, Guru Nanak Dev University, Amritsar 143001, India; 3 Apotex Inc., Toronto, ON M9L 1T9, Canada

**Keywords:** AIE+ESIPT, Cu^2+^, live cells, anticancer, anticounterfeiting

## Abstract

Here, in the present work, a new hydroxybenzothiazole derivative (**HBT 2**) with AIE+ESIPT features was synthesized by Suzuki–Miyora coupling of **HBT 1** with 4-formylphenylboronic acid. The AIE and ESIPT features were confirmed by optical, microscopic (AFM) and dynamic light scattering (DLS) techniques. The yellow fluorescent aggregates of **HBT 2** can specifically detect Cu^2+^/Cu^+^ ions with limits of detection as low as 250 nM and 69 nM. The Job’s plot revealed the formation of a 1:1 complex. The Cu^2+^ complexation was further confirmed by optical, NMR, AFM and DLS techniques. **HBT 2** was also used for the detection of Cu^2+^ ions in real water samples collected from different regions of Punjab. **HBT 2** was successfully used for the bio-imaging of Cu^2+^ ions in live A549 and its anticancer activity was checked on different cancer cell lines, such as MG63, and HeLa, and normal cell lines such as L929. We successfully utilized **HBT 2** to develop security labels for anticounterfeiting applications.

## 1. Introduction

The hydroxyphenyl benzothiazole (HBT) based derivatives have been extensively used in supramolecular chemistry as chemosensors for cations and anions, Refs. [1,2,3] for bioimaging, Refs. [4,5] in medicinal chemistry, Refs. [6,7] as photocatalysts and photosensitizers [8,9], for tracking enzymatic activity [10,11], in latent fingerprinting [12,13,14], easy synthesis [15,16], and for optoelectronic applications [17]. The main reason behind their potential application in various fields relies on their structure which exhibits ESIPT (excited state intramolecular charge transfer) or AIE (aggregation induced enhancement) and in some cases AIE+ESIPT characteristics. In the ESIPT process, lone pairs of electrons on nitrogen atoms, are involved in hydrogen bonding with hydroxyl groups on the phenyl ring [18,19]. AIE-based fluorophores remain non-fluorescent in the solution; however, they become AIE-active in the aggregated state either due to restriction in the rotation or through isolation from the bulk solvent system due to aggregation [20]. Normally, the ESIPT process is inhibited in polar protic solvents such as H_2_O, due to H-bonding. However, organic molecules constructively aggregate in aqueous media and the hydrophobic core allows the ESIPT process to occur. AIE-ESIPT properties of such molecules overcome the ACQ (aggregation caused quenching) phenomena and make it almost *non-emissive* in dilute solution but highly emissive in the aggregated state [21,22]. Recently, Goswami and coworkers reported benzimidazole-based ESIPT active fluorescent chemosensor for the detection of Cu^2+^ ions with LOD as low as 4.22 nM in 90% aqueous solution, in MCF-7 cells and plants [23]. Liang, Zhao and coworkers reported an AIE+ESIPT-based colorimetric Schiff base sensor for the detection of Cu^2+^ ions in aqueous medium [24]. Iyer and coworkers reported HBT based *“turn-on”* fluorometric probe for the detection of Cu^2+^ ions in aqueous medium, real water samples and for bioimaging Cu^2+^ in live cells [25]. However, the literature reports for the detection of Cu^2+^/Cu^+^ using HBT as a potential probe are rare in recent years. To address the problem related to HBT derivatives such as complicated synthesis, working in organic solvents and delayed response time, molecular probes for the selective detection of Cu^2+^ ions in aqueous medium with a lower detection limit are required.

Copper (Cu^2+^) is known as the third most abundant trace element in aerobic organisms and it is involved in many biological pathways [26,27,28,29,30]. Various fundamental physiological functions in the body utilize Cu^2+^ ions, such as in metalloenzymes, respiration, blood formation, transcriptional events, etc. However, the abrupt alteration of Cu^2+^ homeostasis in the body may cause Alzheimer’s disease, anaemia, hypoglycaemia, coronary heart failure, and Wilson’s disease, etc. [31,32,33]. The high level of Cu^2+^ ions is harmful to the human body so the concentration of Cu^2+^ ions in aerobic organisms is regulated at the level of cell, organ and body. The elevated levels of copper are also harmful to aquatic organisms [34]. Copper is also used in machinery, in transportation, and weapons and is an important constituent of white gold in the form of alloy. Various analytical techniques to determine Cu^2+^ in literature includes polarographic methods, iodometric titration, and chemiluminescence method [35,36,37,38]. Apart from these methods, fluorescence-based detection of Cu^2+^ ions have been extensively employed due to its simplicity, high sensitivity, selectivity, rapid response under mild conditions, low cost and potential benefits for a better and deeper understanding of physiological conditions [39,40,41,42].

In continuation of our interest to develop multifunctional chemosensors, recently our group has reported several chemosensors for Cu^2+^ ions and Cu^2+^-based ensembles for the detection of anions, and neutral species in aqueous media, and in live cells and demonstrated their application to develop solid and solution-based kits [43,44,45,46,47,48,49]. Herein, we have reported the synthesis, and characterization of a novel hydroxy benzothiazole (**HBT 2**) based fluorophore featuring ESIPT coupled AIE characteristics for the selective detection of Cu^2+^/Cu^+^ in aqueous media, real water samples and live cells. The AIE+ESIPT characteristics and Cu^2+^ complexation by **HBT 2** have been supported by optical, NMR, DLS and AFM techniques. We also successfully demonstrated the anticounterfeiting and anticancer properties of **HBT 2**.

## 2. Experimental Section

### 2.1. Material and Methods

All reagent-grade chemicals and solvents were purchased from commercial sources and used without further purification. HPLC-grade ethanol and acetonitrile were used. The thin layer chromatography (TLC) was performed on aluminum sheets coated with silica gel 60 F254 (Merck, Darmstadt, Germany). Solvents such as chloroform, ethyl acetate and hexane or their mixture were used to run the TLC. The separation and purification of the products were performed by column chromatography (silica gel 60–120 mesh).

### 2.2. Characterization and Measurements

NMR spectra were recorded on a BRUKER Biospin AVANCE-III FT-NMR HD-500 and JEOL 400 MHz spectrometer operating at 500/400 MHz for ^1^H; 125/100 MHz for ^13^C NMR in CDCl_3_ as a solvent. The peak values were obtained as ppm (δ) and referenced to the TMS in ^1^H NMR and deuterated solvent in ^13^C NMR spectra. Chemical shift (δ) values were reported in ppm, coupling constant (*J* values) in hertz (Hz) and the abbreviations used for splitting patterns are s = singlet, d = doublet, dd = doublet of doublet, t = triplet, q = quartet, m = multiplet. High-resolution mass spectra (HRMS) were recorded with the BRUKER DALTONIK micrOTOFQ11 spectrometer. The Fourier transform infrared (FT-IR) spectra were recorded on the Perkin Elmer 92,035 instrument. The absorbance spectra were recorded on a Cary 5000 UV-VIS-NIR spectrophotometer equipped with a Peltier system to control the temperature. Absorbance spectra were recorded in quartz cells having an appropriate 1 cm path length, 2 nm bandwidth and 140 nm min^−1^ scan rate. Fluorescence spectra were recorded with the Shimadzu RF-6000 spectrofluorophotometer. Quartz glass cuvettes of 10 mm thickness were used for recording emission data. A Nano-ZS Malvern Instrument was employed for performing dynamic light scattering (DLS) analysis. AFM images were captured using Tosca 400 instrument made by Anton Paar under ambient conditions. Before DLS/AFM measurements each stock solution was pre-filtered through a Millipore membrane filter (Acrodisc syringe filter, 0.2 µm Supor membrane). Thermal gravimetric analysis was carried out on the HITACHI STA7200 instrument.

### 2.3. UV-Visible and Fluorescence Studies

The stock solution for the photophysical studies of **HBT 2** (0.001 M) was prepared in CH_3_CN. HEPES buffer (0.1 M, pH 7.2) was prepared using deionized water. A stock solution was diluted using CH_3_CN and HEPES buffer for the photophysical measurements. Further, stock solutions of analytes (0.1 M) were prepared and diluted in deionized water. For determining the binding constant and stoichiometry of the Cu^2+^ complex, the Benesi–Hildebrand equation, Job plot and SPECFIT 32 software were used.

### 2.4. Sample Preparation for Cu^2+^ Analysis in Real Samples

We collected real water samples from different locations in Punjab and these water samples were used to make the HEPES buffer solution. The solution of Cu^2+^ ions was also prepared in these water samples. **HBT 2** (0.5 µM) in HEPES buffer–CH_3_CN (9:1, *v*/*v* pH 7.2) solution was taken in a cuvette and then Cu^2+^ ions were added to simulate a polluted water sample. Then, a recovery experiment was performed on the Cu^2+^ spiked samples. All of the experiments were conducted in triplicate. The fluorescence intensities of these real samples were recorded and compared with standard calibration curves.

### 2.5. Anticancer Studies (MTT Assay)

The cytotoxic potential of **HBT 2** towards HeLa, MG63 and L929 cell lines was determined using an MTT assay. The cells were cultured at the standard density of 8 × 103 cells/well in 96 well microplates in 100 μL of DMEM medium. These cultured cells were then incubated for 24 h to allow the adherence of cells. After incubation for 24 h, cells were treated with various concentrations of **HBT 2**. On completion of another 12 h, we added 20 µL of 3-(4,5-dimethylthiazol-2-yl)-2,5-diphenyltetrazolium bromide (MTT) dye solution in each well and incubation was continued for an additional 2 h for measuring the ability of viable cells to reduce it into purple coloured formazan. Subsequently, we removed the supernatant MTT solution and then added 100 µL of dimethyl sulphoxide (DMSO) to dissolve the intracellular insoluble, purple-coloured formazan. Then absorbance was taken at 310 nm using a multi-well plate reader (BioTek Synergy HT, BioTek, Winooski, VT, USA).

Cell viability = (Absorbance of treated sample/Absorbance of untreated control) × 100(1)

The growth inhibition percentage was expressed by using the following equation

% Growth inhibition = 100% viability.(2)

### 2.6. The Cell Imaging

A549 cells were incubated in an in vitro incubator under 5% humidified CO_2_ at 37 °C in DMEM containing 10% fetal bovine serum and antibiotic antimycotic solution. Later on, the DMEM was removed from culture plates and a trypsin solution was added followed by washing the culture palates 2–3 times with phosphate buffer saline (PBS). The cells were divided into four groups (i) blank containing A549 cells only; (ii) blank containing derivative **HBT 2** (1 µM) only in 99% PBS buffer (1% CH_3_CN); (iii) **HBT 2** (1 µM) + Cu^2+^ ions (10 equivalents); and (iv) **HBT 2** (1 µM) + Cu^2+^ ions (20 equivalents). All the cells were incubated in triplicates and Cu^2+^ treated wells were incubated for 30 min. After washing three times with PBS, the fluorescence images were captured under a fluorescence microscope.

### 2.7. Synthesis of Derivative ***HBT 1***

To the solution of 5-bromo salicylaldehyde (1.0 g, 4.9 mmol) and 2-aminothiophenol (0.62 g, 4.97 mmol) in ethanol (10 mL), 10 µL of H_2_O_2_ (30% *v*/*v*) and 20 µL HCl (2N) were added. The mixture was stirred for 2–3 h at 70–80 °C. The progress of the reaction was checked by TLC. After completion of the reaction, the mixture was cooled to room temperature and precipitates were filtered and washed with ethanol to afford compound **HBT 1** as a yellow solid, (1.3 g, 85.5%). R*_f_* = 0.35 (Hexane: CHCl_3_, 70: 30, *v*/*v*); ^1^H NMR (500 MHz, CDCl_3_, 25 °C) δ 12.55 (s, 1H), 8.00 (d, *J* = 8.1 Hz, 1H), 7.93 (d, *J* = 8.0 Hz, 1H), 7.79 (d, *J* = 2.4 Hz, 1H), 7.57–7.50 (m, 1H), 7.47–7.42 (m, 2H), 7.00 (d, *J* = 8.8 Hz, 1H) ppm; ^13^C NMR (125 MHz, CDCl_3_, 25 °C) δ 167.80, 157.04, 151.66, 135.34, 132.63, 130.50, 126.95, 125.97, 122.39, 121.65, 119.83, 118.36, 111.04 ppm; IR (ATR):ν (in cm^−1^) = 3056.4, 2952.1, 2773.1, 2109.7, 1997.9, 1878.6, 1781.7, 1572.9, 1476, 1267.3, 1088.4, 976.6, 872.2, 775.3, 626.2, 514.4, 454.7.

### 2.8. Synthesis of Derivative ***HBT 2***

To a solution of **HBT 1** (1.0 g, 3.27 mmol) in toluene (10 mL), Pd(PPh_3_)_4_ (0.566 g, 0.48 mmol) was added under a N_2_ stream. Subsequently, 4-formylphenylboronic acid (0.588 g, 3.9 mmol) and Na_2_CO_3_ (0.699 mg, 6.5 mmol) dissolved in ethanol:water (1:1) was added to the reaction mixture and stirred at 80℃ for 4–5 h. The solvent was evaporated under a rotary evaporator and water was added. The progress of the reaction was checked by TLC. The residue was extracted with CHCl_3_. The CHCl_3_ layers were dried over sodium sulfate and solvent was rotary evaporated. The crude mixture was column chromatographed on SiO_2_ using CHCl_3_: Hexane (70:30) as eluent to afford **HBT 2** as pale-yellow solid (880 mg, 80.8%); R*_f_* = 0.77 (CHCl_3_); ^1^H NMR (500 MHz, CDCl_3_) δ 12.73 (s, 1H), 10.07 (s, 1H), 8.02 (d, *J* = 8.1 Hz, 1H), 7.98 (d, *J* = 8.2 Hz, 2H), 7.95–7.91 (m, 2H), 7.76 (d, *J* = 8.2 Hz, 2H), 7.67 (dd, *J_1_* = 8.6, *J_2_ =* 2.1 Hz, 1H), 7.54 (t, *J* = 7.7 Hz, 1H), 7.44 (t, *J* = 7.6 Hz, 1H), 7.22 (d, *J* = 8.6 Hz, 1H) ppm; ^13^C NMR (125 MHz, CDCl_3_) δ 191.84, 168.88, 158.42, 151.75, 145.98, 134.99, 132.58, 131.67, 131.23, 130.46, 127.12, 127.06, 126.93, 125.87, 122.34, 121.64, 118.74, 117.21, ppm; FTIR (ATR):ν (in cm^−1^) = 3705, 2981.9, 2832.8, 2743.3, 2363.1, 2117.1, 1923.3, 1692.2, 1483.5, 1386.6, 1304.6, 1170.4, 820.0, 745.5, 492.0; HRMS: calcd: 332.0701, found: 332.0738.

## 3. Results and Discussion

### 3.1. Synthetic Design

**Scheme 1:**
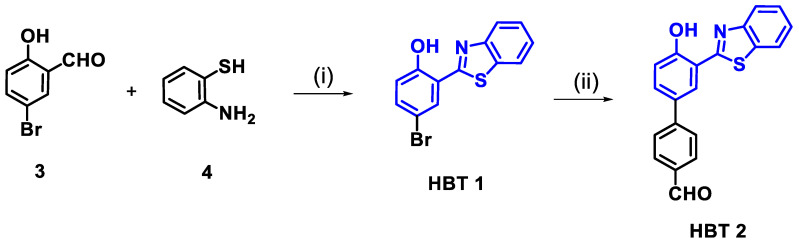
Synthesis of derivatives **HBT 1** and **HBT 2**; *Reagents and conditions*: (i) ethanol, 70 °C, 3 h, then H_2_O_2_/HCl (ii) 4-formylphenylboronic acid, Na_2_CO_3_, Pd(PPh_3_)_4_, toluene, C_2_H_5_OH:H_2_O (1:1), 80 °C, 5 h.

**HBT 2** containing benzothiazole at the 2-position and 4-formyl phenyl at the 4-position of the phenol ring was synthesized in two steps. In the first step, compound **HBT 1** was synthesized by condensation (Schiff-base) reaction between 5-bromosalicylaldehyde (**3**) and 2-aminothiophenol (**4**) in ethanol at 70 °C, followed by in-situ intramolecular cyclization reaction catalyzed by the H_2_O_2_/HCl mixture. In the second step, a Suzuki–Miyora coupling reaction between 4-formylphenylboronic acid and **HBT 1** in toluene afforded **HBT 2** at a 80% yield (Scheme 1) [46]. The characterization of compounds **HBT 1**, and **HBT 2** was accomplished with ^1^H, ^13^C NMR, IR and high-resolution mass spectrometry (HRMS) techniques (Figures S1 and S2 from Supplementary Materials). The observance of ^1^H NMR signals at 10.07 ppm (s, -CHO) and 12.73 ppm (s, -OH), along with other aromatic proton signals in the range δ = 8.02–7.22 ppm confirmed the structure of **HBT 2**. HRMS data showed a mass peak at *m*/*z* 332.0738 (M+H)^+^. The fluorescence spectrum of **HBT 2** showed an emission maximum located at 550 nm in the solution or solid state. Thermogravimetric analysis (TGA) of **HBT 2** showed stability up to 280 °C and then a drastic mass loss of 85% occurred up to 400 °C (Figure S3 from Supplementary Materials). On the other hand, **HBT 1** is stable only up to 200 °C and complete mass loss (78%) occurred at 280 °C (Figure S4 from Supplementary Materials).

### 3.2. Photophysical Studies to Support AIE+ESIPT Features in ***HBT 2***

**Figure 1:**
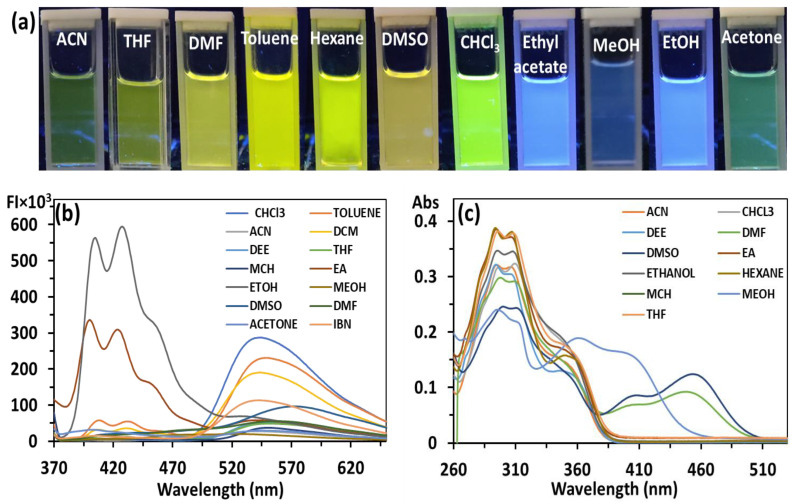
(**a**) Photographs (under 365 nm UV-illumination); (**b**) emission and (**c**) absorbance spectrum of **HBT 2** (10 μM) recorded in various solvents of different polarity. Photographs were taken at concentration 10 μM of **HBT 2**.

**Figure 2:**
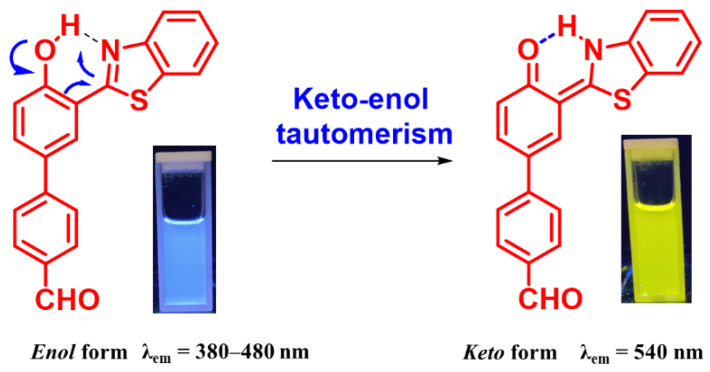
The general mechanism for *keto–enol* tautomerism in **HBT 2**.

In the literature, the aggregation induced emission (AIE) process is normally associated with a restriction in intramolecular rotation (RIR) in a solid state or in an aggregated state [19]; however, many reports are available where the occurrence of an excited state intramolecular proton transfer (ESIPT) process in an aggregated state results in an enhancement in emission intensity commonly known as ESIPT coupled AIE [20]. In **HBT 2**, there is a possibility of ESIPT and AIE process. The ESIPT process was first validated by recording the absorbance and emission spectra of **HBT 2** (10 µM) in solvents of different polarities (Figure 1). In non-polar solvents, **HBT 2** showed absorption bands at 295, and 310 nm and the shoulder band at 340 nm, whereas in polar solvents the appearance of an additional band between 390 and 460 nm was also observed. This additional red-shifted band in polar solvents could be attributed to either a hydrogen bond cluster or due to the formation of an anion via deprotonation of the -OH group (Figure 1c). Similarly, the emission spectra of **HBT 2** in non-polar solvents such as CHCl_3_, DCM and THF showed an emission band at 540 nm (λ_ex_ = 360 nm), but in polar (protic) solvents a new band in the range of 380–480 nm was observed. This emission band at 380–480 nm could be attributed to the *enol* form while the bathochromically shifted emission band at 540 nm could be attributed to *keto* form arising due to the ESIPT process in **HBT 2**. The color of the **HBT 2** solution in polar solvents is blue and in non-polar solvents, it is yellow, which is in good agreement with the assumption of the existence of *enol*-tautomer in polar solvents, and *keto*-tautomer in non-polar solvents (Figure 1a and Figure 2).

The rationalization of the *enol*–*keto* equilibrium in **HBT 2**, was also performed using a B3LYP/6-31G set in Gaussian 09 software. Analysis of the optimized structures shows that the HOMO and LUMO of *keto*-form are more stable than the *enol*-form by −0.53 and −0.24 eV. Time-dependent density functional theory revealed that the lowest energy absorption transitions correspond to 340 nm (Figure S5 from Supplementary Materials).

**Figure 3:**
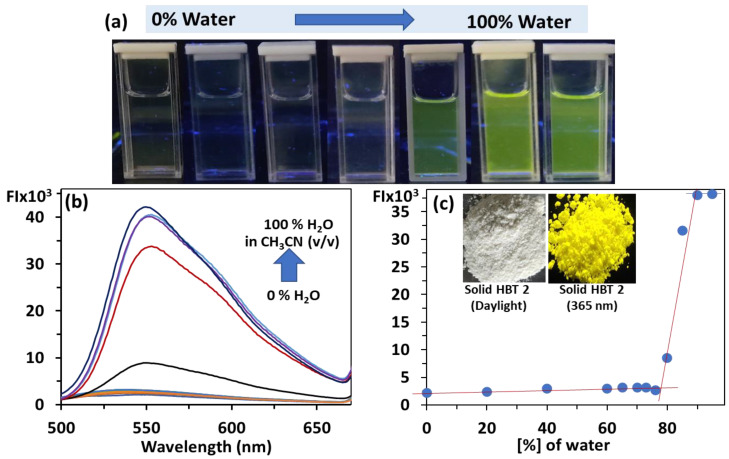
(**a**) Photographs (under 365 nm UV-illumination); (**b**) emission spectra (0.5 µM) of **HBT 2** recorded in different fractions of water (0–100%) in CH_3_CN and (**c**) plot of fluorescence intensity versus fraction of water [inset of (**c**)] photograph of **HBT 2** (solid) in daylight and **HBT 2** mixed with silica under 365 nm UV lamp.

To validate the existence of AIE in **HBT 2**, we have recorded the optical spectra of **HBT 2** in different binary aqueous mixtures where we have continuously varied the fraction of water (bad solvent) in CH_3_CN (good solvent) (Figure 3). In CH_3_CN, **HBT 2** (0.5 μM) exhibits a very weak broad emission band between 500 and 600 nm. Upon incremental addition of the water fraction from 0–70%, no change in the emission spectrum was observed and the fluorescence remained low [φ_f_ = 0.022, measured against quinine sulfate (φ_f_ = 0.54) as standard solution in H_2_SO_4_]. When the fraction of water was further increased from >70–100% in CH_3_CN, a drastic boost in fluorescence intensity (~30 times) at 550 nm was observed (φ_f_ = 0.096) due to the formation of fluorescent nanoaggregates. The colorless solution of **HBT 2** in ˂70% H_2_O–CH_3_CN mixture indicates the existence of **HBT 2** in a non-aggregated state, whereas the observance of bright greenish-yellow-colored nanoaggregates in 70–99% water indicates the existence of **HBT 2** in the aggregated state (Figure 3a,c).

Similarly, UV-vis spectra of **HBT 2** in CH_3_CN and H_2_O–CH_3_CN (0–100%) mixture were also recorded. **HBT 2** showed an absorbance band at 295 nm. In addition to 0–80% water in CH_3_CN, it showed a decrease in the molar absorptivity of the absorbance band at 295 nm. However, on further addition of water fraction (>80%) in CH_3_CN, the absorption spectra broadened and the molar absorptivity of the absorbance band increased (Figure S6 from Supplementary Materials). These spectroscopic changes are associated with the AIE+ESIPT mechanism and the explanation is as follows. In the 50% H_2_O–CH_3_CN mixture, the -OH group remains H-bonded with water molecules (protic solvent). As the water fraction increases, **HBT 2** starts organizing itself to make supramolecular nanoaggregates. In an aggregated state, the access of water molecules decreases and ESIPT becomes operative in the aggregated state of **HBT 2** and fluorescence at 550 nm increases. 

### 3.3. Validation of Aggregation in ***HBT 2*** Using AFM and DLS Techniques

**Figure 4:**
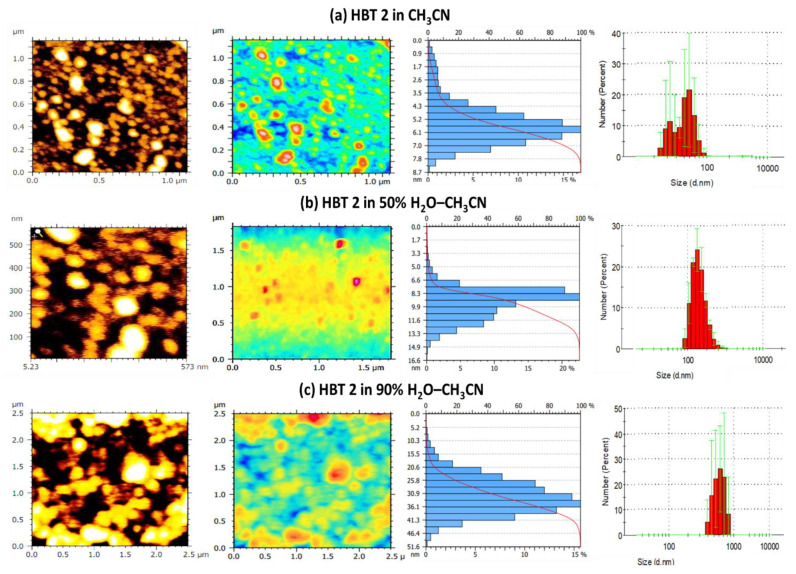
AFM micrographs, electron density profile, histogram showing size of aggregates of **HBT 2** (1 µM) and DLS bar graph of **HBT 2** (0.5 µM) in (**a**) CH_3_CN; (**b**) 50% HEPES buffer:CH_3_CN and (**c**) 90% HEPES buffer:CH_3_CN solution.

To shed more insight on the aggregation process and morphology of the aggregates of **HBT 2** upon the addition of H_2_O to CH_3_CN, we have performed atomic force microscopic (AFM) imaging of **HBT 2** in pure CH_3_CN, 50% HEPES buffer–CH_3_CN and 90% HEPES buffer–CH_3_CN solution. The AFM micrographs of **HBT 2** (1 µM) in CH_3_CN showed spherical aggregates of the size of 0.9–7.8 nm uniformly distributed all over the surface. However, the size of the aggregates of **HBT 2** increased to 1.7–14.9 nm in 50% H_2_O–CH_3_CN solution. On the other hand, in the 90% H_2_O–CH_3_CN solution, the size of the aggregates of **HBT 2** increased to 10.3–47.4 nm. Interestingly, the morphology of the aggregates of **HBT 2** in CH_3_CN, 50% HEPES buffer–CH_3_CN and 90% HEPES buffer–CH_3_CN remains spherical in nature and no change in morphology of these aggregates was observed as we move from CH_3_CN to 90% HEPES buffer–CH_3_CN solution (Figure 4a–c).

We further confirmed this aggregation process by performing dynamic light scattering (DLS) analysis. The hydrodynamic diameter of aggregates of **HBT 2** (0.5 µM) in the CH_3_CN (where it exists as a molecularly dissolved state) was 92.6 nm (polydispersity index = 0.66). In a 1:1 (CH_3_CN: HEPES buffer) solution, the size of the aggregates was increased to 330.7 nm (polydispersity index = 0.63), which further increased to 615.5 nm (polydispersity index = 0.639) in 9:1 (HEPES buffer: CH_3_CN) solution (Figure 4a–c). This increase in the size of the aggregates of **HBT 2** could be attributed to the aggregation process upon the gradual addition of water. Moreover, the high value of the polydispersity index (˃0.6) indicates the presence of aggregates of different sizes in the solution.

### 3.4. Application of ***HBT 2*** for Detection of Cu^2+^/Cu^+^ Ions

We expect that different metal ions may interact with the hydroxy (-OH) group and nitrogen (N) atoms of **HBT 2** and the complexation of **HBT 2** with metal ions may inhibit or reverse the AIE+ESIPT phenomena. So, we recorded the optical response of **HBT 2** in the presence of various metal ions such as Li^+^, Na^+^, NH_4_^+^, K^+^, Zn^2+^, Co^2+^, Ni^2+^, Pb^2+^, Fe^2+^, Fe^3+^, Ba^2+^ Cu^+^ and Cu^2+^ in 9:1 (HEPES buffer:CH_3_CN, pH 7.2, *v*/*v*) solution. The **HBT 2** showed strong quenching of fluorescence in the presence of Cu^+^ and Cu^2+^ ions only, while the other competing metal ions did not show any interference (Figure S7 from Supplementary Materials). As shown in Figure S7 from Supplementary Materials, **HBT 2** showed a response only towards Cu^+^ and Cu^2+^ ions, so next, we performed fluorescence titration with Cu^2+^/Cu^+^ ions.

**Figure 5:**
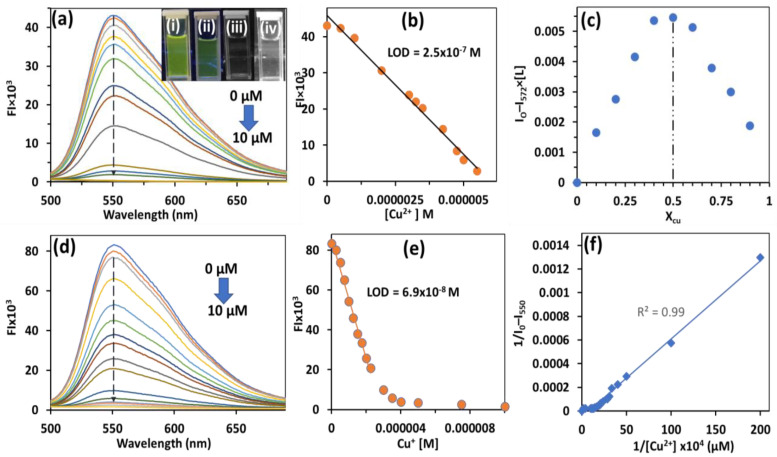
(**a**,**d**) Emission spectrum of **HBT 2** (0.5 μM) upon addition of Cu^2+^ and Cu^+^ ions recorded in 90% HEPES buffer–CH_3_CN, pH 7.2; (**b**,**e**) plot of fluorescence intensity versus conc. of Cu^2+^ ions for calculating the limit of detection; (**c**) Job’s plot showing 1:1 stoichiometry between **HBT 2** and Cu^2+^ ions; and (**f**) B–H plot between I/I_o_–I_550_ and 1/Cu^2+^ ion; [Inset of (**a**)] Photographs (under 365 nm UV lamp) showing quenching of fluorescence of (**i**) **HBT 2** upon addition of (**ii**) 5, (**iii**) 10 and (**iv**) 20 equivalents of Cu^2+^ ions.

Upon addition of 0–20 equivalents of Cu^2+^ and Cu^+^ ions, the emission spectrum of **HBT 2** showed a regular decrease in fluorescence at 520 nm with concomitant fluorescence color change from bright yellow to colorless (Figure 5a,d). On further addition of Cu^2+^ and Cu^+^ ions (˃20 equivalents), no change in the fluorescence spectrum was observed and the plateau was achieved. It may be noted that plateau was achieved at lower concentrations for Cu^+^ (six equivalents) ions compared to Cu^2+^ (10 equivalents) ions. This decrease in fluorescence could be attributed to the complexation of Cu^+^ or Cu^2+^ ions with oxygen and nitrogen atoms of **HBT 2** which results in the inhibition of the ESIPT process and fluorescence quenching was observed. Alternatively, the binding of Cu^2+^ or Cu^+^ ions with **HBT 2** may disturb the aggregation of **HBT 2** and consequently, the inhibition of AIE could lead to quenching of the fluorescence. The lowest limit of detection was found to be 69 nM (R^2^ = 0.99) and 250 nM (R^2^ = 0.99) for Cu^+^ and Cu^2+^, respectively (Figure 5b,e). The Job’s plot between **HBT 2** and Cu^2+^ ions showed 1:1 binding stoichiometry (Figure 5c). The Benesi–Hildebrand (B-H) plot was used to find the binding constant of complexation between **HBT 2** and Cu^2+^ ions and it was found to be 7.14 × 10^4^ M^−1^ (Figure 5f). The non-linear regression curve fitting analysis using SPECFIT programmer revealed 1:1 stoichiometry with a binding constant of log β = 5.829 (for Cu^+^) and log β = 5.454 (for Cu^2+^). The absorbance spectrum of **HBT 2** did not show any variation upon the addition of Cu^2+^ ions in a 90% H_2_O–CH_3_CN mixture.

To develop metal–complex based displacement assay for the ‘*turn-on*’ detection of anions, we added different anions such as I^−^, Br^−^, CN^−^, ClO_4_^−^, ATP, AMP, ADP, UTP, UDP, UMP, GMP, CMP, S_2_O_4_^−^, SCN, HSO_4_^−^, HSO_3_^−^, NO_3_^−^, F^−^, H_2_S and P_2_O_4_^−^ to the solution of **HBT 2**-Cu^2+^ complex in 90% HEPES buffer–CH_3_CN, pH 7.2 solution. However, upon the addition of these anions even at higher concentrations (50 equivalents) to the **HBT 2**–Cu^2+^ complex, insignificant change was observed (Figure S8 from Supplementary Materials). This could be ascribed to the fact that the complexation of Cu^2+^ ion with **HBT 2** is strong and the anion may not be able to sequester it from the binding site of **HBT 2**.

### 3.5. NMR, DLS and AFM Studies of ***HBT 2*** with Cu^2+^ Ions

**Figure 6:**
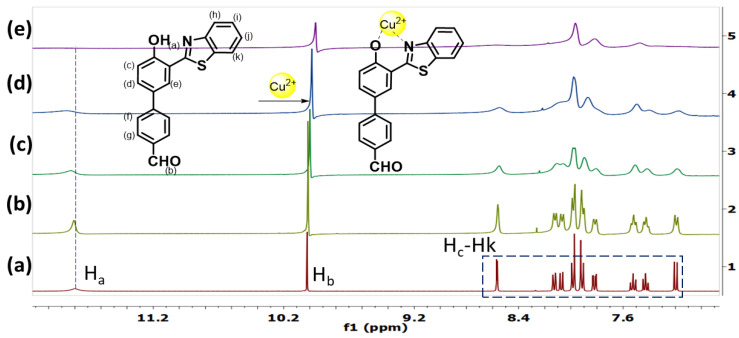
^1^H NMR spectra (partial) of **HBT 2** (5 mM); (**a**) in the absence and presence of (**b**) 1; (**c**) 3; (**d**) 5 and (**e**) 10 equivalents of Cu^2+^ ions recorded in DMSO(*d*_6_).

To obtain further insight into the mechanism of complexation between **HBT 2** and Cu^2+^ ions, we have performed the NMR titration in the absence and presence of Cu^2+^ ions (Figure 6). Upon addition of 1, 3, 5 and 10 equivalents of Cu^2+^ ions in **HBT 2**, the -OH peak of **HBT 2** at δ = 11.8 ppm broadened and then disappeared whereas the sharp doublet of H_c_ at δ = 8.565 ppm shifted up-field to δ = 8.547 and then broadened. Furthermore, the ^1^H NMR analysis showed an up-field shift of the aldehyde proton at δ = 10 ppm to 9.96 ppm whereas signals of the aromatic protons H_d_–H_k_ become upfield shifted and merged with each other with concomitant broadening upon gradual addition of Cu^2+^ ions. These NMR changes could be due to the complexation of Cu^2+^ with -OH and -N of the **HBT 2** which caused the deprotonation of -OH. The broadening of the signals may be due to the paramagnetic effect of the Cu^2+^ ions bound to **HBT 2**.

**Figure 7:**
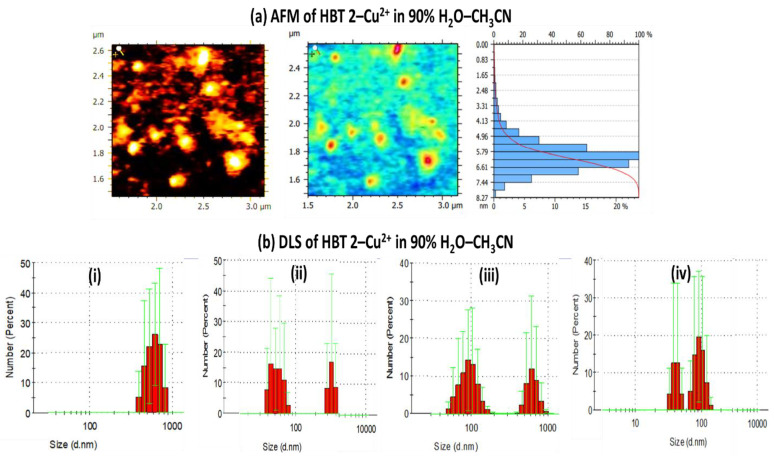
(**a**) AFM images, electron density profile, aggregate size-based bar graph of **HBT 2** (1 µM), in the presence of Cu^2+^ ions recorded in 9:1 (HEPES buffer:CH_3_CN) solution and (**b**) DLS analysis of (**i**) **HBT 2** (0.5 μM) upon addition of (**ii**) 5, (**iii**) 10 and (**iv**) 20 equivalents Cu^2+^ ions recorded in 9:1 (HEPES buffer: CH_3_CN) solution.

We have also performed the atomic force microscopic (AFM) analysis of **HBT 2** in the absence (Figure S9 from Supplementary Materials) and the presence of Cu^2+^ ions. The AFM micrographs of the aggregates of **HBT 2** (1 µM) in 9:1 (HEPES buffer: CH_3_CN) showed spherical aggregates of the size of 10.3–47.4 nm. Upon the addition of 20 equivalents of Cu^2+^ ions, the size of the aggregates decreased to 2.5–8 nm, however, the spherical morphology remains the same (Figure 7a). We further confirmed the de-aggregation process by performing a DLS analysis. The hydrodynamic diameter of **HBT 2** (0.5 µM) in the aggregated state (HEPES buffer: CH_3_CN, 9:1) was 615.5 nm (polydispersity index = 0.66). Upon the addition of five equivalents of Cu^2+^, it was decreased to 192.91 nm (polydispersity index = 0.63), which further decreased to 91.28 nm (polydispersity index = 0.639) and 50.75 nm (polydispersity index = 0.491) on adding 10 and 20 equivalents of Cu^2+^ ions, respectively (Figure 7b). These results further support the disaggregation process in **HBT 2** upon the addition of Cu^2+^ ions which consequently diminished the AIE phenomena.

### 3.6. Application in Determination of Cu^2+^ in Real Water Samples

To evaluate whether the **HBT 2** can be used for the determination of Cu^2+^ ions in real water samples, we carried out the experiment of standard addition and recovery method by taking water samples from different areas of Punjab (India) such as Amritsar, Bathinda (rural, town), Muktsar and Faridkot. Different concentrations of Cu^2+^ ions in the range between (1–8 µM) were spiked into these water samples and then subsequently **HBT 2** was added. The fluorescence spectra were recorded for each sample. The obtained fluorescence values at 550 nm for each real water sample spiked with Cu^2+^ were compared with the calibration curve and data are presented in Table 1 and Figure S10 from Supplementary Materials. The percentage recovery of Cu^2+^ ions from these samples ranged from 93–105% demonstrating the practical utility of **HBT 2** for the detection of the Cu^2+^ ions in real water samples.

### 3.7. Application of ***HBT 2*** as Anti-Counterfeiting Labels

**Figure 8:**
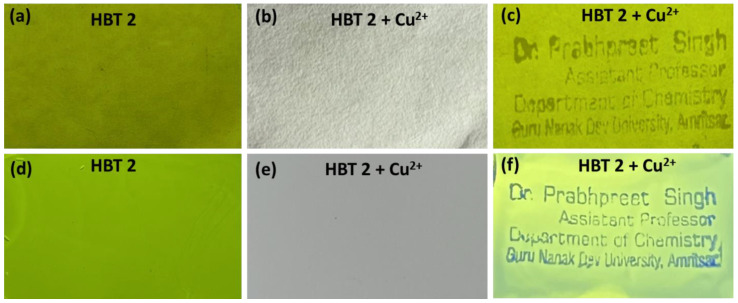
Photographs of **HBT 2** (under 365 nm UV lamp) alone; **HBT 2** after stamping with Cu^2+^ ink under daylight and 365 nm UV lamp taken on (**a**–**c**) filter papers and (**d**–**f**) TLC strips for developing anticounterfeiting labels.

**HBT 2** is highly emissive in the solid state so we further used it for developing anti-counterfeiting labels. For this purpose, we first coated **HBT 2** (1 mM) on filter paper and TLC strips by dip and dry method using CHCl_3_ solution. The fluorescent colour of the coated **HBT 2** on filter paper and TLC strips was not visible to the naked eye or in daylight. However, on illumination under 365 nm UV lamps, it glows by emitting yellow-green fluorescence (Figure 8a,d).

Subsequently, we stamped several alphabets (using the stamp of the principal Investigator) on **HBT 2** coated filter paper and TLC strips by using a stamp pad immersed in Cu^2+^ ions (1 mM) solution as a security ink. Under normal daylight, no alphabets were seen on these **HBT 2** coated filter paper or TLC strips (Figure 8b,e). However, upon illumination of these **HBT 2** coated filter paper and TLC strips with UV light of 365 nm, the dark coloured alphabets of the stamp can be clearly seen (Figure 8c,f). This could be ascribed to the quenching of the greenish-yellow fluorescence of the **HBT 2** by Cu^2+^ ions. Moreover, by using a high concentration of Cu^2+^ ions (5 mM), it is possible to see the alphabets of stamps with the naked eye in daylight as green colour alphabets (Figure S11 from Supplementary Materials).

### 3.8. Intracellular Detection of Cu^2+^ in A549 Cells

**Figure 9:**
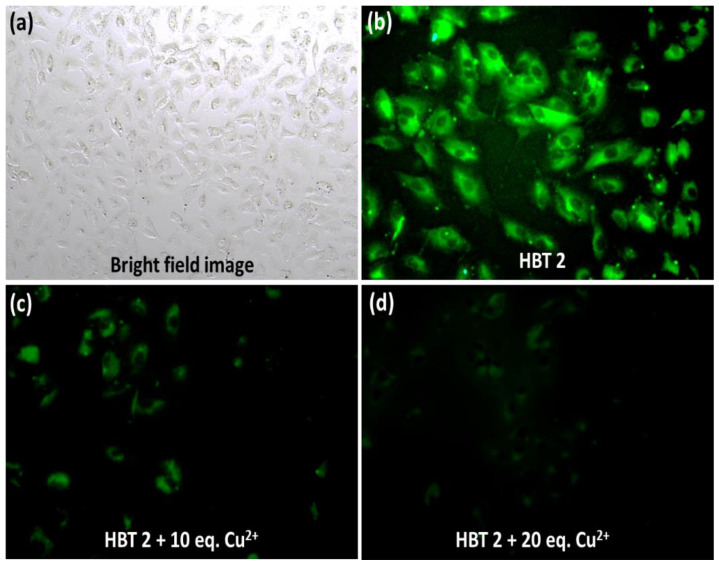
(**a**) Bright field image; (**b**) Fluorescence image (at 10×) of A549 lung cancer cells incubated with **HBT 2** (1 μM); Fluorescence image of A549 cells incubated with **HBT 2** followed by incubation with (**c**) 10 equivalents and (**d**) 20 equivalents of Cu^2+^ ions.

To further validate the application of **HBT 2** for the real-time monitoring of Cu^2+^ ions in live cells under physiological conditions, fluorescence microscopic imaging experiments were performed. The A549 cells were first pre-treated with **HBT 2** (1 μM) for 30 min and then further incubated with different concentrations of Cu^2+^ ions (10 and 20 equivalents) for 30 min at 37 °C and imaged under inverted fluorescence microscopy. As shown in Figure 9b, the A549 cells, which were incubated with **HBT 2** (1 μM), showed strong green fluorescence inside the cytoplasm which indicates that **HBT 2** has good cell membrane permeability. The bright field images showed no change in the morphology of the cells. Upon the exogenous addition of Cu^2+^ ions (10 and 20 equivalents) to the cells pre-treated with **HBT 2**, the green fluorescence was quenched in a concentration-dependent manner (Figure 9c,d). These results are similar to the results obtained in the solution phase study. These observations suggested the potential application of **HBT 2** in bioimaging and the detection of Cu^2+^ ions in live cells.

### 3.9. Anticancer Activity of ***HBT 2***

**Figure 10:**
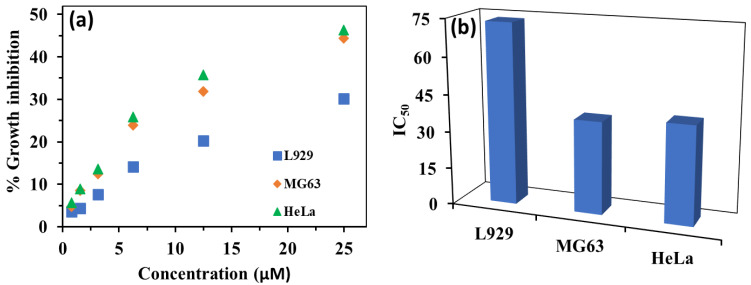
(**a**) Effect of increasing dose of **HBT 2** (0–25 µM) on viability of normal and cancer cell lines and (**b**) Bar graph of IC_50_ values.

To check the cytotoxicity of **HBT 2**, we performed the MTT assay on different cell lines such as HeLa cells (cervical cancer cells), MG 63 cells (bone cancer cells) and L929 cells (normal fibroblast cells) to confirm the cellular death response. The cells were treated with various concentrations of **HBT 2** (0.78, 1.56, 3.125, 6.25, 12.5 and 25 µM) for HeLa, MG 63 and L929 cells followed by incubation for 24 h. We observed that **HBT 2** showed cytotoxicity (IC_50_ = 37.3 ± 1.12 μM) and selectivity index = 1.96 against the MG 63 cells and IC_50_ = 39.58 ± 1.25 µM and selectivity index = 1.85 against HeLa cells in comparison to normal L929 cells (IC_50_ = 73.46 ± 1.28 µM). It has also been found that the cell viability has been reduced on treatment with **HBT 2** in a dose-dependent manner (Figure 10 and Figure S12 from Supplementary Materials).

## 4. Conclusions

In conclusion, we have synthesized a hydroxybenzothiazole (HBT) based derivative **HBT 2** possessing AIE+ESIPT properties in aqueous medium. **HBT 2** showed excellent selectivity for Cu^+^/Cu^2+^ ions in 90% aqueous medium. The mechanism of interaction involves disaggregation of **HBT 2** aggregates in the presence of Cu^2+^ ions supported by optical, NMR, AFM and DLS techniques. We have successfully demonstrated applications of **HBT 2** for (i) determination of Cu^2+^ concentrations in real water samples; (ii) bioimaging and detection of Cu^2+^ ions in A549 cells; (iii) anticancer activity against MG-63, HeLa cells and (iv) anti-counterfeiting labels using **HBT 2** coated filter paper and TLC strip and a solution of Cu^2+^ as a security ink.

## Figures and Tables

**Table 1:** Determination of Cu^2+^ ions in spiked real water samples taken from different areas of Punjab (India) using **HBT 2** in HEPES buffer–CH_3_CN (9:1, *v*/*v*, pH 7.2) solution.

Technique Used	Samples Taken from	Cu^2+^ Added (µM)	Cu^2+^ Found (µM)	% Age Recovery
Fluorescence	Amritsar	2.6	2.5	98.03
		3.4	3.5	102.94
		5.6	5.5	99.09
		7.7	7.5	99.29
	Bathinda (Rural)	3.5	3.0	95.38
		4.5	4.5	95.23
		6.2	6.0	100.00
		7.9	7.5	95.54
	Bathinda (Town)	1.6	1.5	95.23
		3.2	3.0	93.75
		4.7	4.5	96.77
		7.6	7.5	99.33
	Muktsar	1.6	1.5	96.15
		2.7	2.5	94.33
		4.6	4.5	97.82
		7.5	7.5	100.00
	Faridkot	1.7	1.5	90.90
		2.5	2.5	99.00
		4.5	4.5	100.00
		6.2	6.5	105.69

## Supplementary Materials

References from [22,50,51,52,53,54,55] are cited from supplementary materials. The supporting information can be downloaded at: https://www.mdpi.com/article/10.3390/molecules27227678/s1, the original spectral and photophysical data (Figures S1–S12 and Tables S1).

## Sample Availability

Samples of the compounds **HBT 1** and **2** are available from the authors.
