# Supplementary material for: AIE+ESIPT Active Hydroxybenzothiazole for Intracellular Detection of Cu2+: Anticancer and Anticounterfeiting Applications
Source: Molecules. 2022 Nov 8;27(22):7678. doi: 10.3390/molecules27227678 (PMC9699452; doi:10.3390/molecules27227678)
Supplement: Supplementary file 1 [file molecules-27-07678-s001.zip › molecules-1972934-supplementary.pdf]

molecules

MDPI

Supplementary Materials

AIE+ESIPT Active Hydroxybenzothiazole for Intracellular Detection of Cu^{2+}: Anticancer and Anticounterfeiting Applications

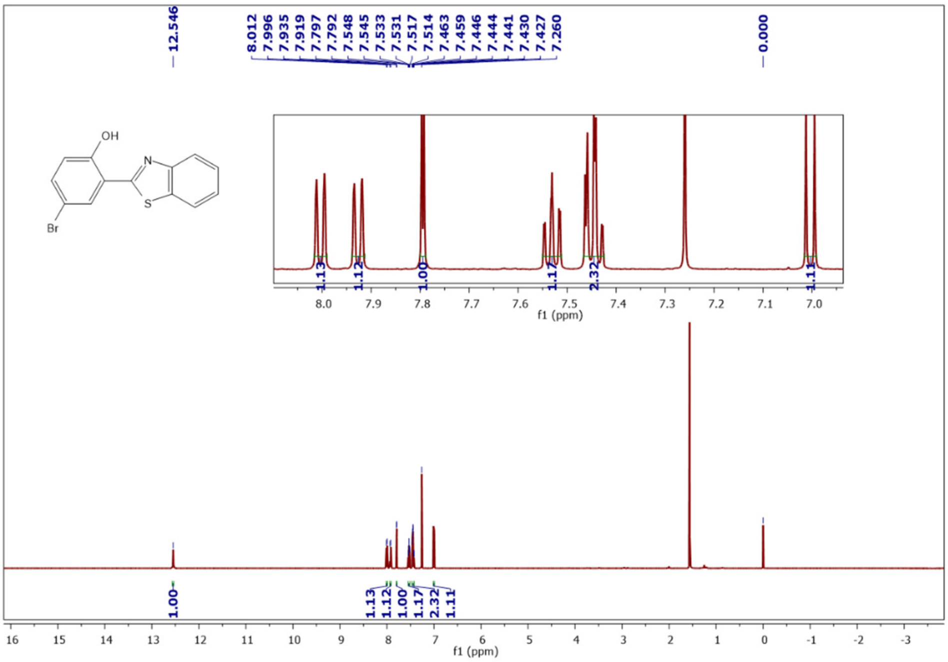

(**a**)

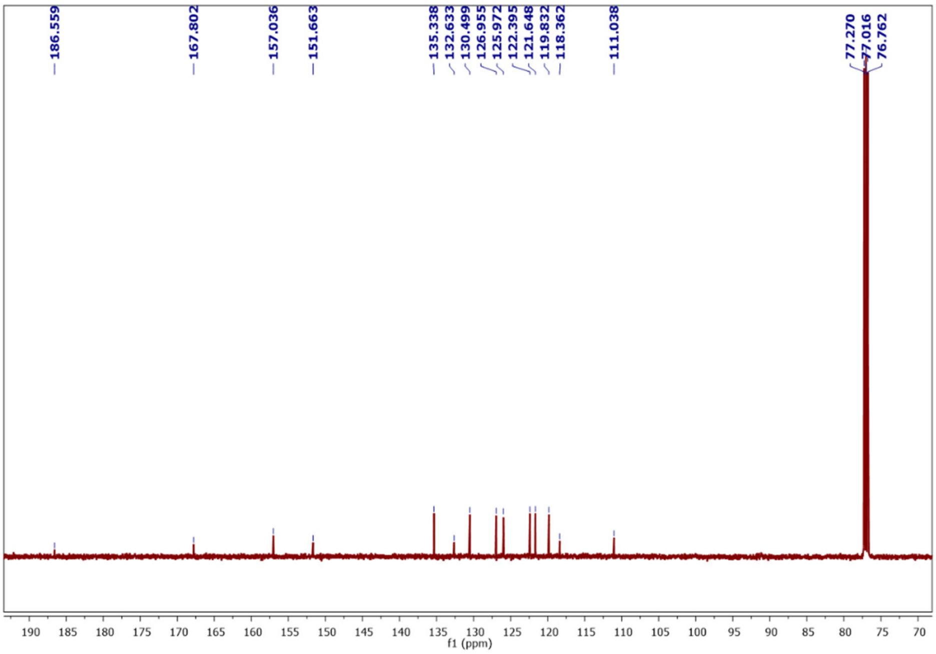

(**b**)

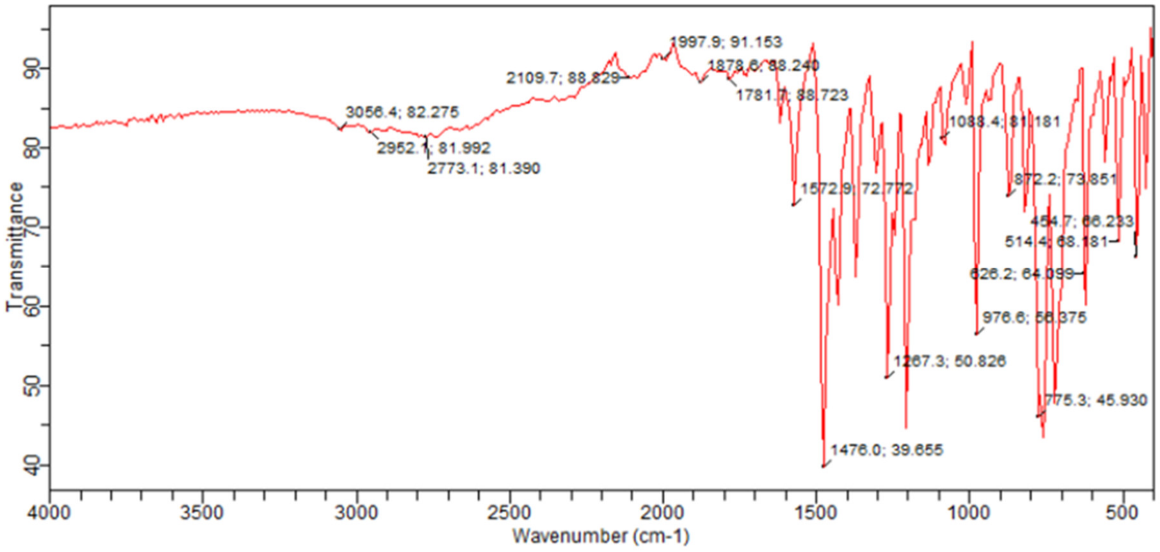

(**c**)

Figure S1. a: ^{1}H NMR spectra of probe '**HBT 1**'. **b:** ^{13}C NMR spectra of probe '**HBT 1**'. **c:** FT-IR spectra of probe **HBT 1.**

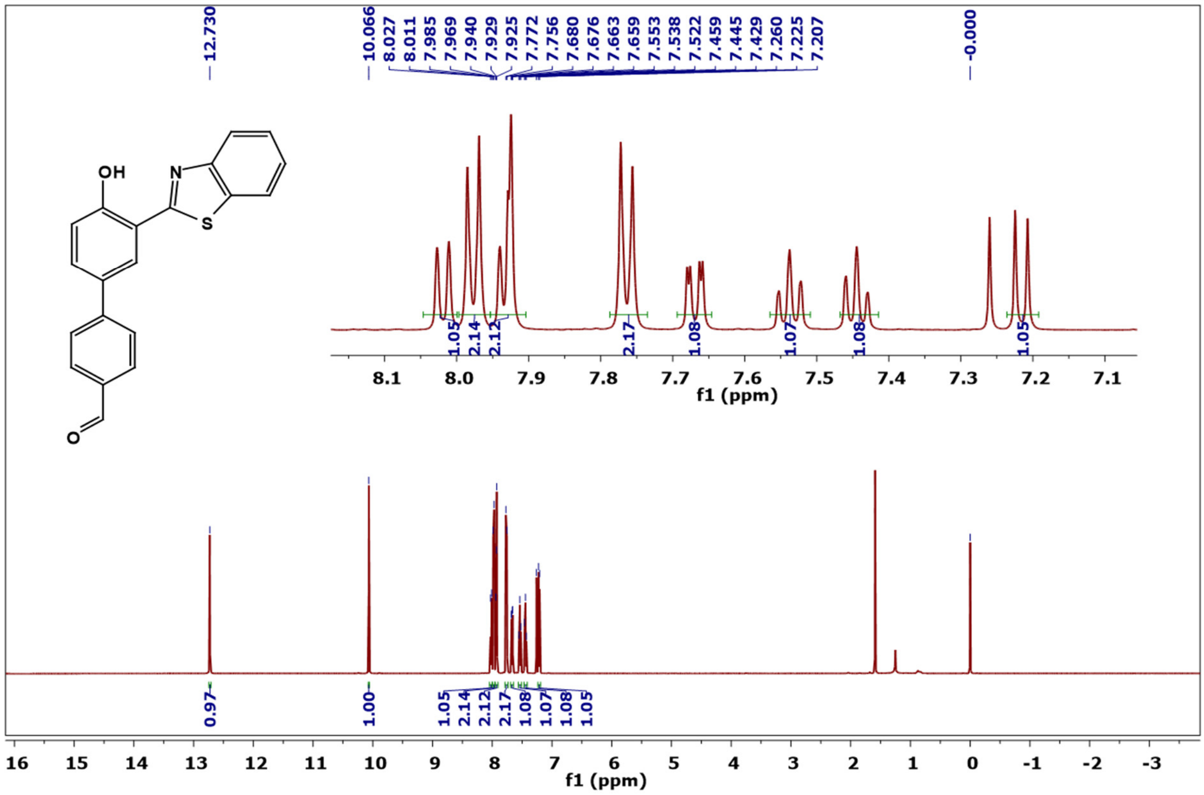

(**a**)

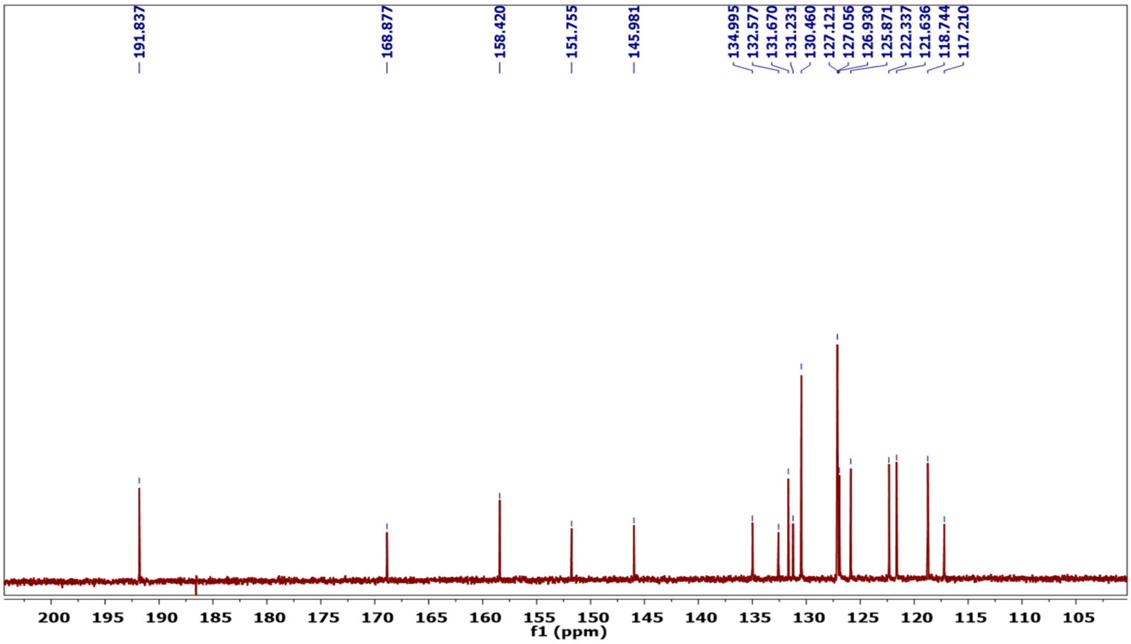
191.837
168.877
158.420
151.755
145.981
134.995
132.577
131.670
131.231
130.460
127.121
127.056
126.930
125.871
122.337
121.636
118.744
117.210
200 195 190 185 180 175 170 165 160 155 150 145 140 135 130 125 120 115 110 105
f1 (ppm)

(**b**)

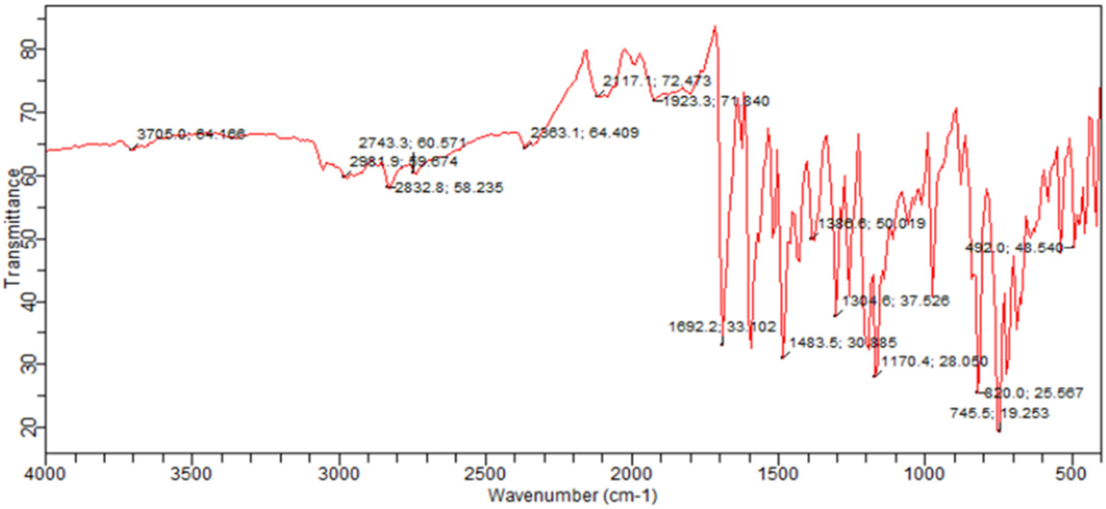
Transmittance
Wavenumber (cm-1)
3705.0; 64.168
2743.3; 60.571
2981.9; 59.674
2832.8; 58.235
2363.1; 64.409
2117.1; 72.473
1923.3; 71.840
1692.2; 33.102
1483.5; 30.885
1304.6; 37.526
1170.4; 28.050
820.0; 25.567
745.5; 19.253
492.0; 48.540

(**c**)

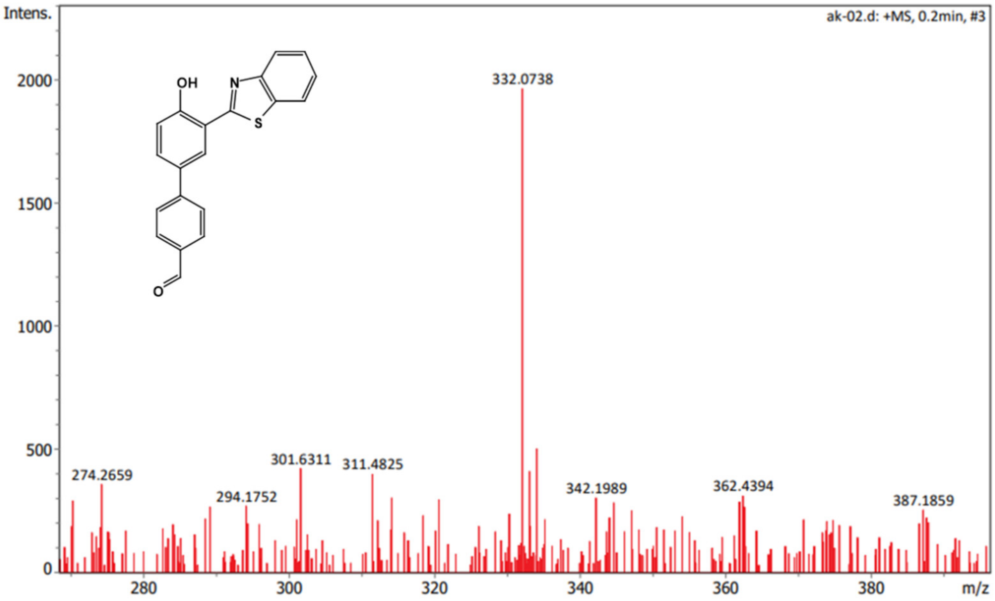

(**d**)

Figure S2. a: ^{1}H NMR of HBT 2.. b: ^{13}C NMR of HBT 2. **c**: FT-IR spectra of **HBT 2. d**: Mass spectra of HBT 2.

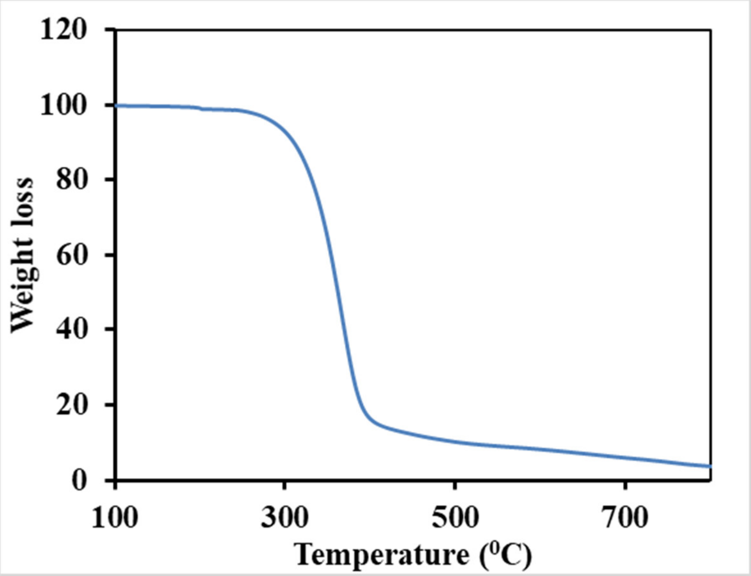

Figure S3. TGA spectra of HBT 2.

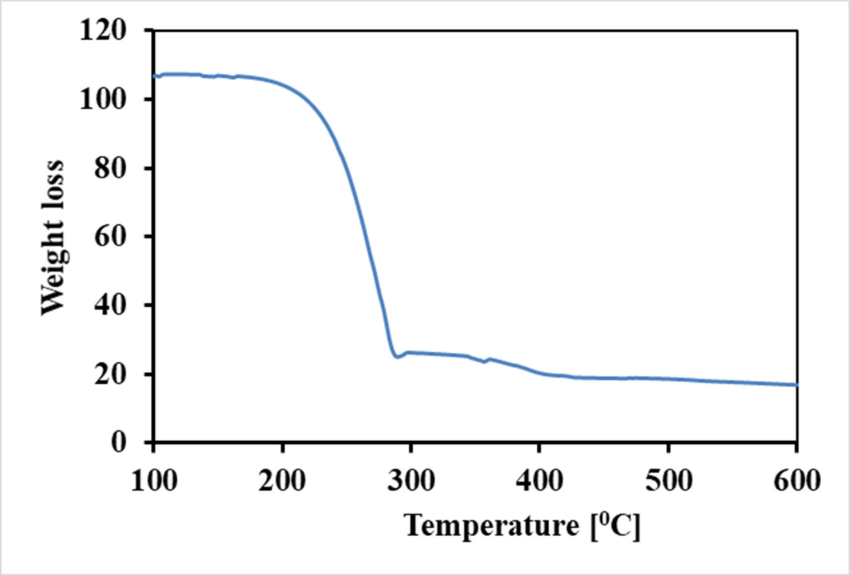

Figure S4. TGA spectra of HBT 1.

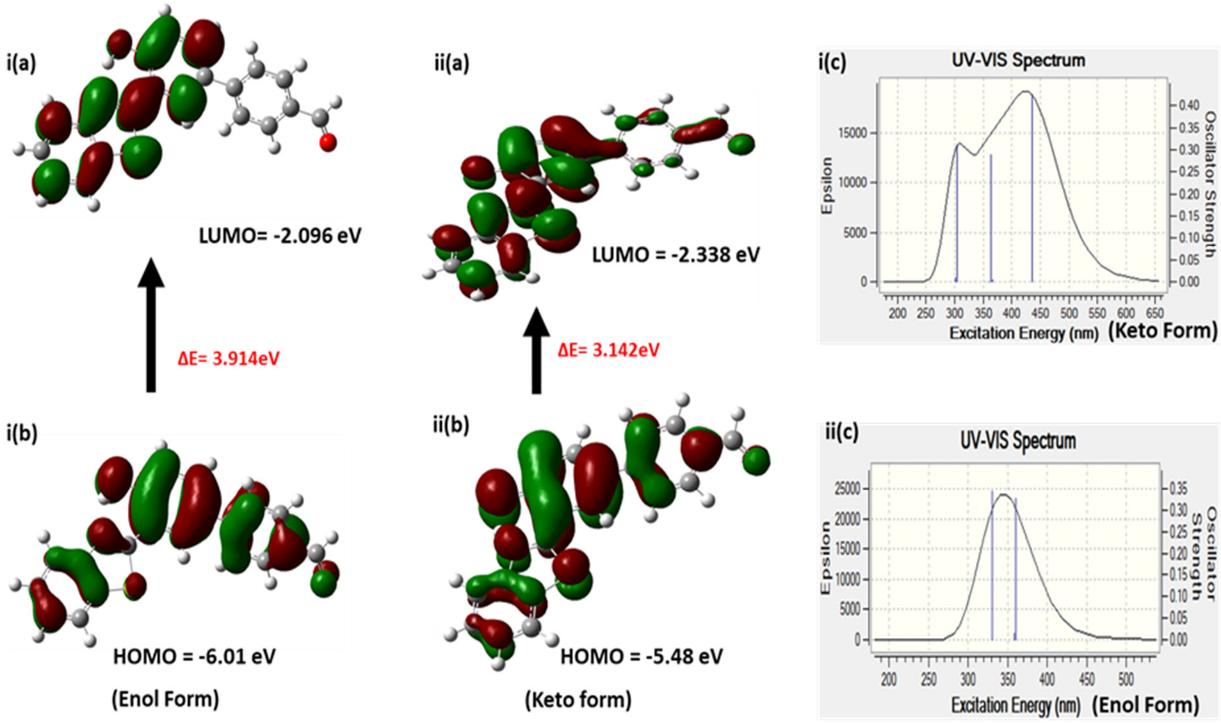

Figure S5. (a) Contour structures of possible conformation (*keto* and *enol*) of HBT **2** with their energies (HOMO and LUMO) calculated using the DFT (B3LYP/6-31G*) level.

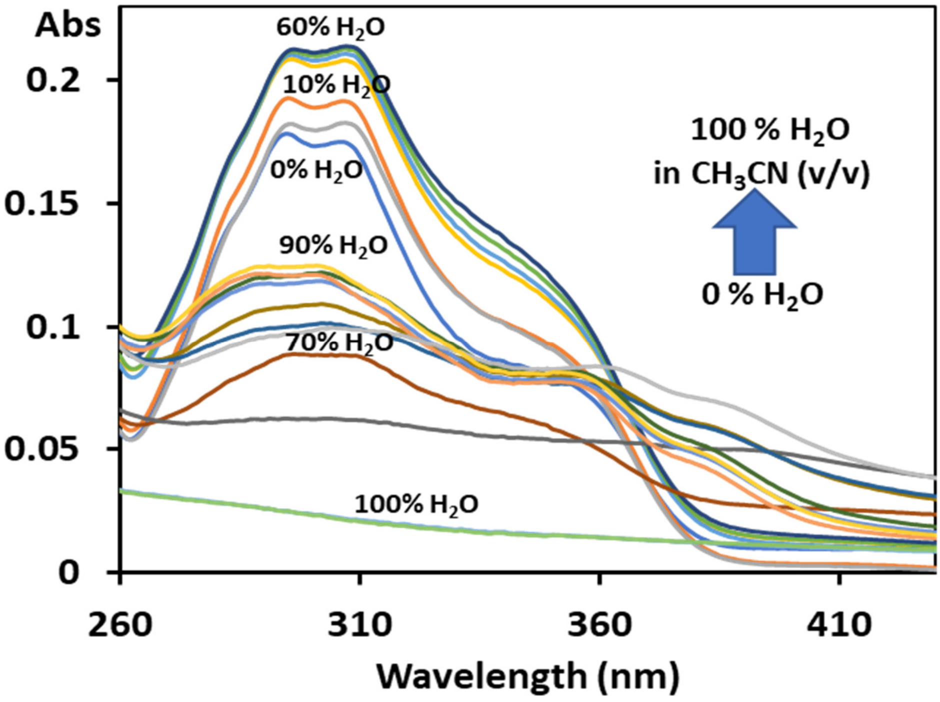

Figure S6. Absorbance spectra (10 μM) of **HBT 2** recorded in different fractions of water (0–100 %) in CH3CN.

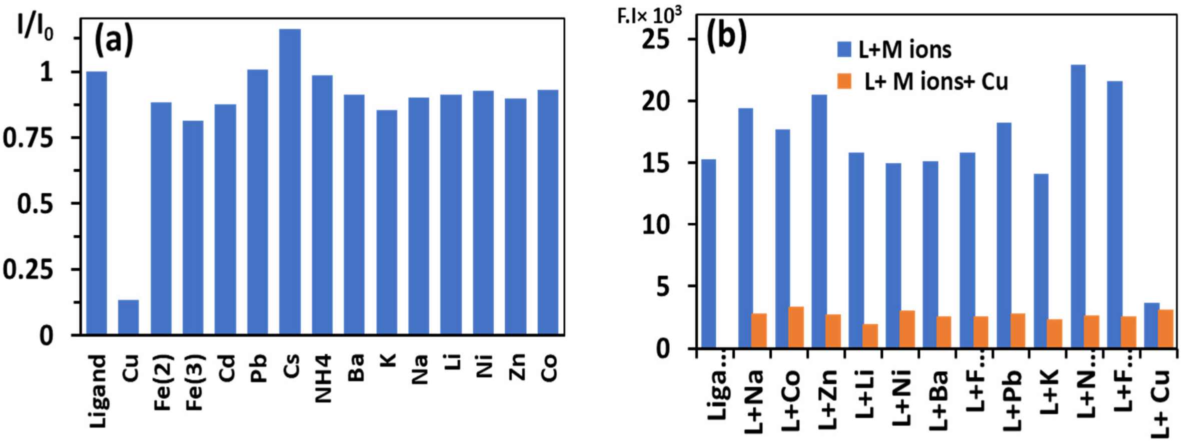

Figure S7. (a) Selectivity experiment of HBT **2** for Cu^{2+} ions; (b) Interference experiment of **HBT 2** for detection of Cu^{2+} ions in the presence of various metal ions recorded in 90% HEPES buffer–CH3CN, pH 7.2. Ligand = **HBT 2.**

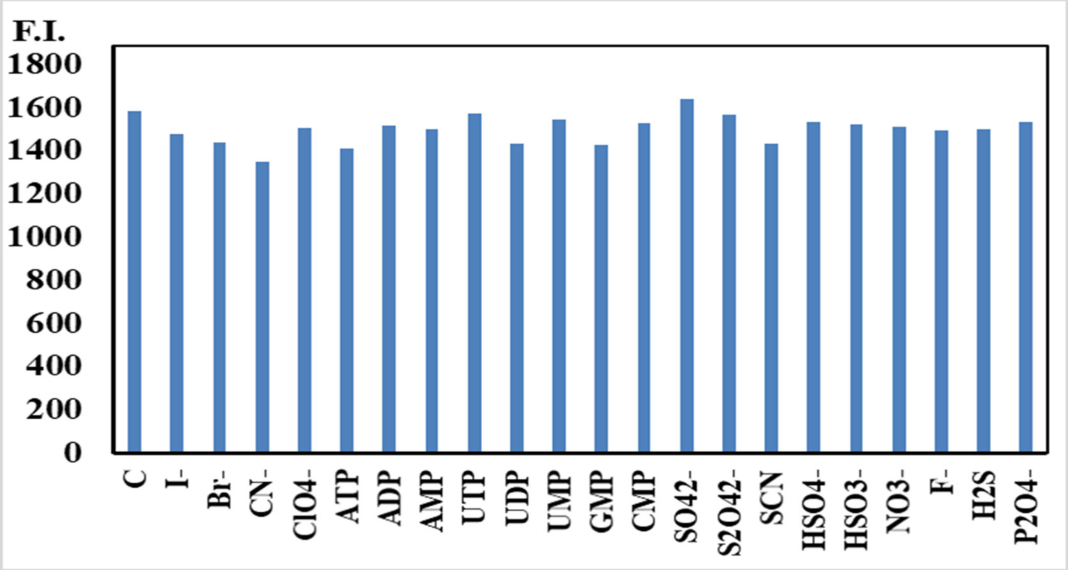

Figure S8. Bar graph upon addition of different anions in **HBT 2**–Cu^{2+} complex recorded in 90% HEPES buffer–CH_3CN, pH 7.2.

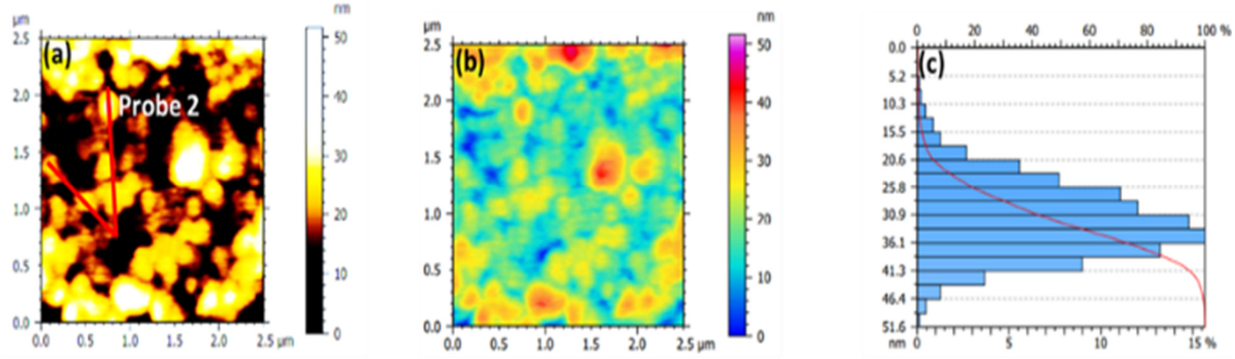

Figure S9. AFM images, electron density profile, aggregate size-based bar graph of **HBT 2** (1 μM), in the absence of Cu^{2+} ions recorded in 9:1 (HEPES buffer:CH_3CN) solution.

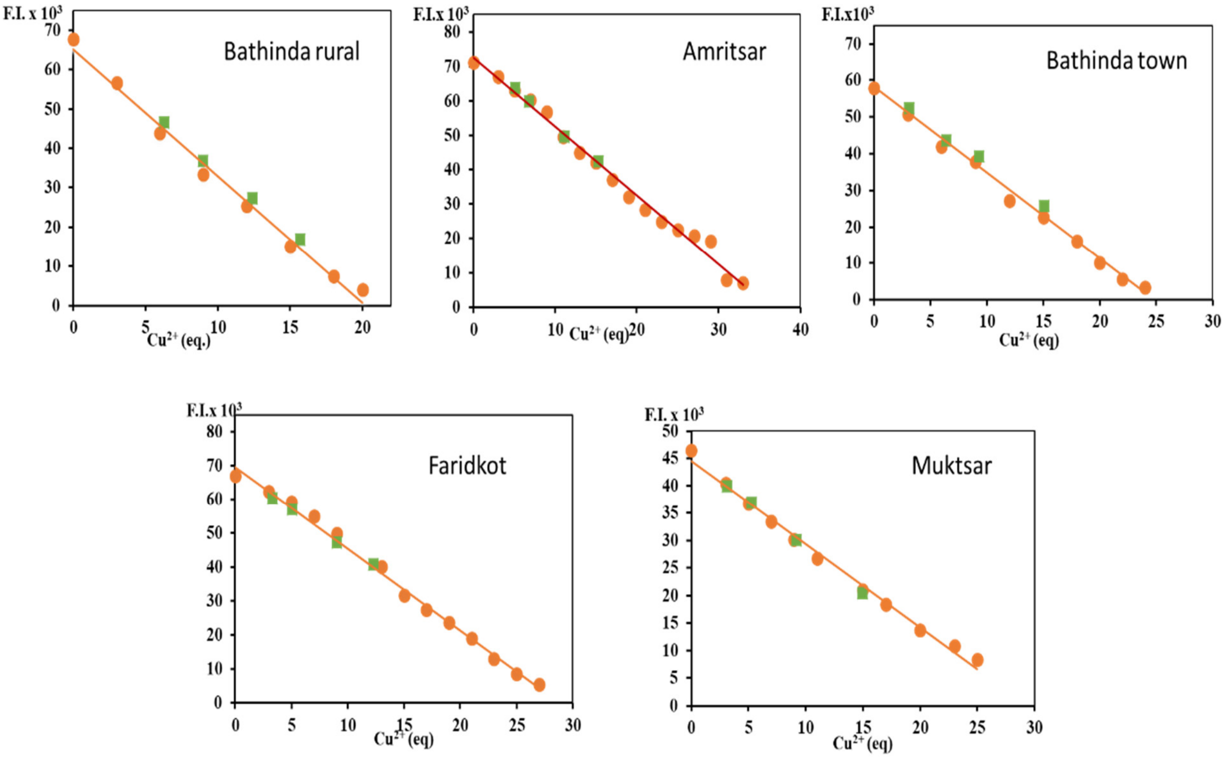

Figure S10. Calibration curves (for calculating the percent recovery) for Cu^{2+} spiked samples taken from different areas of Punjab (India).

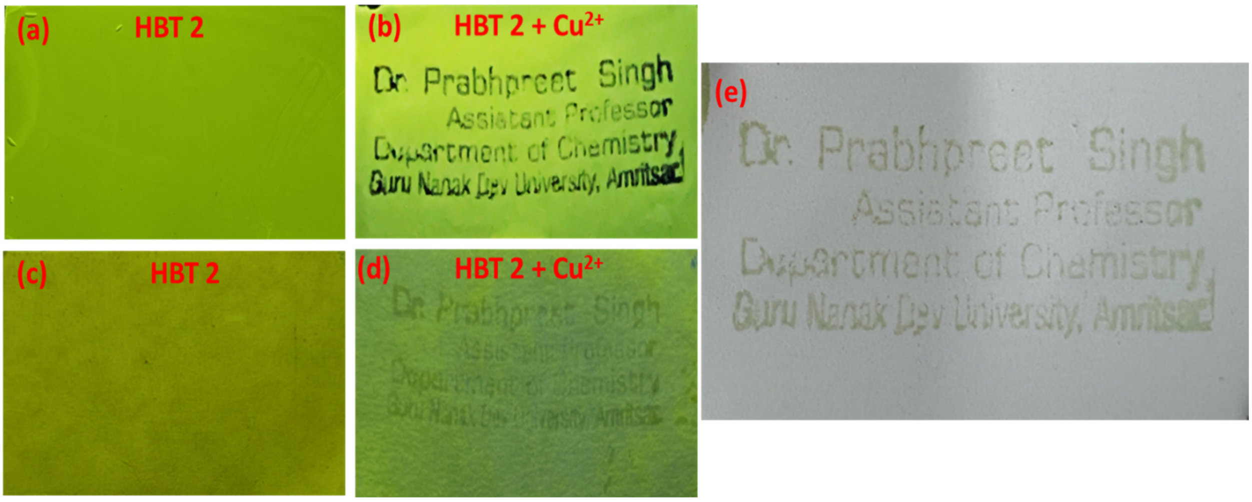

Figure S11. Photographs of TLC and Filter-paper strips coated with (a, c) **HBT 2**, (b, d) stamped with alphabets using Cu^{2+} as security ink (under 365 nm UV lamp) (e) **HBT 2** stamped with alphabets using Cu^{2+} as security ink (under daylight) for anticounterfeiting applications.

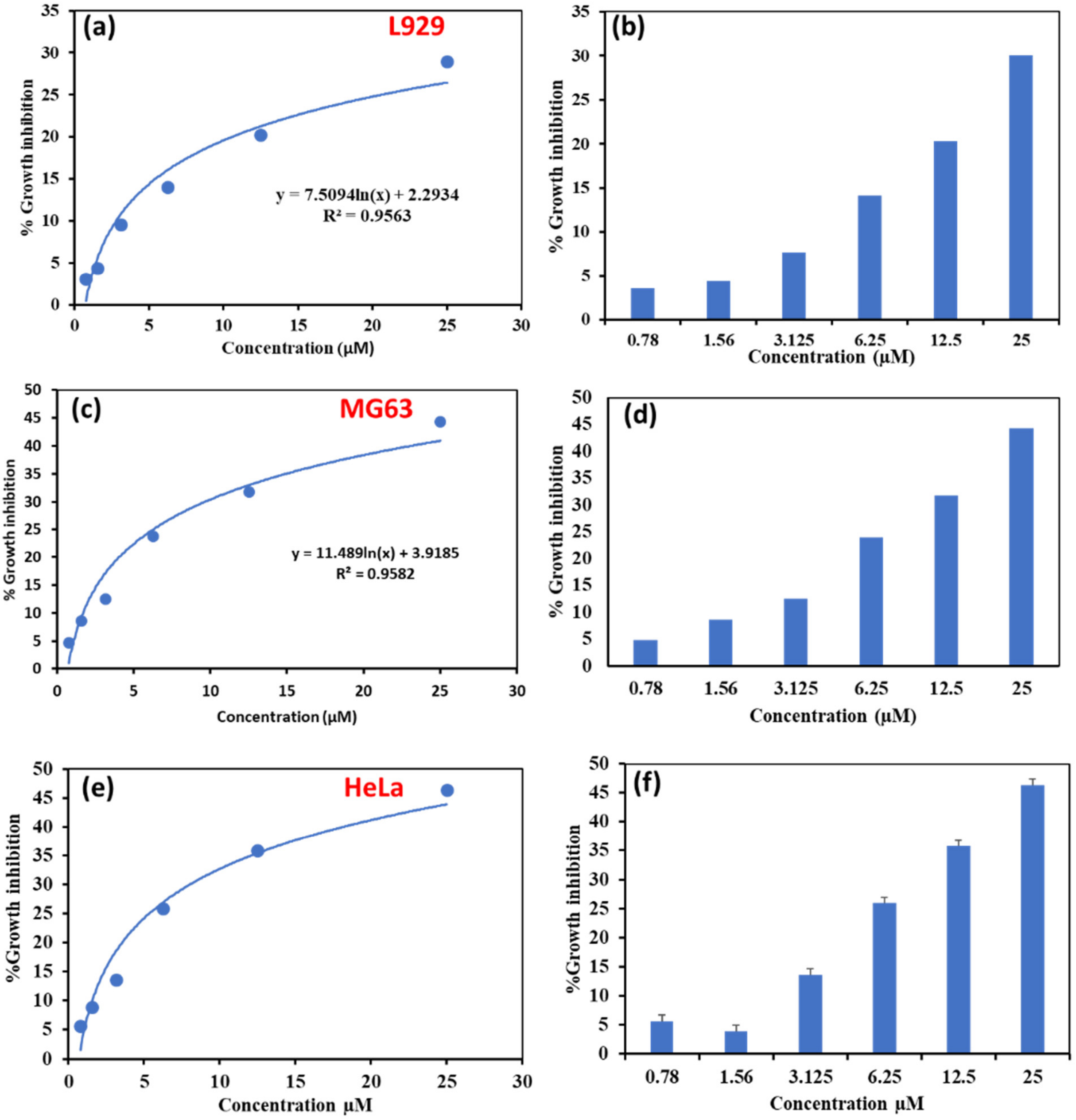

Figure S12. MTT assay on different cell lines (a, b) L929 (c, d) MG63 (e, f) HeLa cells.

Table S1. Report comparison of **HBT 2** with other HBT Derivatives.

Probe structure	Analytes	LOD(M)	Solvent system	Application	Reference
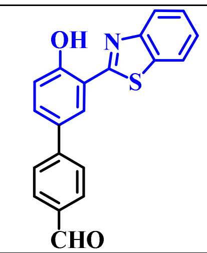	**Cu^{2+}**	0.25 µM	CH_3CN: H_2O (1:9)	Real water sample analysis, Live cell, Anticancer and anticounterfeiting	This work
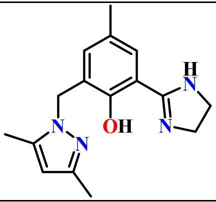	**Cu^{2+}**	28.5 nM	EtOH: H_2O (1:9)	NO	[1]
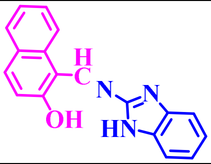	**Cu^{2+}**	0.8 µM	EtOH: H_2O (1:1)	NO	[2]
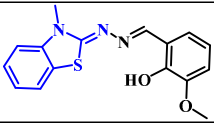	**Cu^{2+}**	0.37 µM	CH_3CN	Solid state detection	[3]
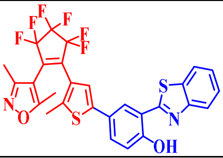	**Cu^{2+}**	Not mentioned	CH_3CN	NO	[4]
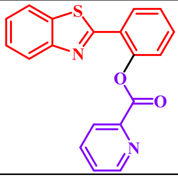	**Cu^{2+}**	16.1 n M	CH_3CN: H_2O (1:4)	NO	[5]
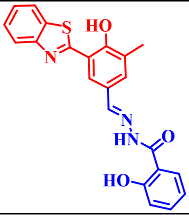	**Cu^{2+}**	1.35 µM	DMF: H_2O (3:7)	Paper strip	[6]
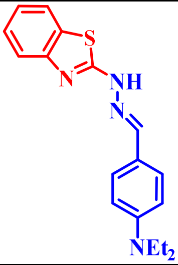	**Cu^{2+}**	0.7 µM	CH_3CN: H_2O (3:1)	NO	[7]

References:

1. N. Guchhait, A. Bhattacharyya, S. C. Makhal and S. Mandal *New J. Chem.*, 2019, **43**, 15087-15096
2. S. Goswami, R. Bag, Y. Sikdar, S. Sahu, C. D. Mukhopadhyay and M. Drew, *J. Photochem. & Photobiol.*, 2022, **431,** 114006
3. C. Kim, J. S. Heo and B. Suh, *J. Chem. Sci.* 2022, **43**, 134
4. S. Pu, R. Wang, X. Dong, G. Leo and P. Leo, *Tetrahedron*, 2016, **72**, 2935-2942
5. Y. Han, C. Yang, Y. Chen, K. Wu, T. Wei, J. Wang and S. Zhang, *Anal. Methods*, 2015, **7**, 3327
6. Z. Xing, J. Li, J. Wang, Z. Tian, D. Wu and Y. Xiang, *Spectrochimica Acta Part A: Mol. and Biomol. Spec.* 2020, **235**, 11831
7. K. Chansaenpak, J. Nootem, R. Daengngern, C. Sattayanon, W. Wattanathana, S. Wannapaiboon and P. Rashatasakhon, *J. Photochem. & Photobio.*, 2021, **415**, 113318
